# Cost‐effectiveness analysis of postoperative surveillance for stage IV colorectal cancer in Japan: An economic modeling study

**DOI:** 10.1002/ags3.12906

**Published:** 2025-01-13

**Authors:** Fumio Tsukamoto, Shunsuke Tsukamoto, Takeharu Kato, Hiroshi Nagata, Yasuyuki Takamizawa, Konosuke Moritani, Yusuke Kinugasa, Minoru Esaki, Yukihide Kanemitsu, Ataru Igarashi

**Affiliations:** ^1^ Department of Colorectal Surgery National Cancer Center Hospital Tokyo Japan; ^2^ Department of Gastrointestinal Surgery, Graduate School of Medicine Institute of Science Tokyo Tokyo Japan; ^3^ Department of Hepatobiliary and Pancreatic Surgery National Cancer Center Hospital Tokyo Japan; ^4^ Department of Health Economics and Outcomes Research, Graduate School of Pharmaceutical Science The University of Tokyo Tokyo Japan

**Keywords:** colorectal cancer, cost‐effectiveness analysis, metastasis, quality‐adjusted life years, surveillance

## Abstract

**Background:**

The optimal postoperative surveillance strategy after curative resection in patients with stage IV colorectal cancer remains unclear. The present study aimed to assess the cost‐effectiveness of postoperative surveillance strategies recommended by the various academic societies for stage IV colorectal cancer after curative resection.

**Methods:**

This economic evaluation used a Markov state‐transition model to compare the cost‐effectiveness of postoperative surveillance programs proposed in guidelines published by the American Society of Clinical Oncology, American Society of Colon and Rectal Surgeons, European Society for Medical Oncology, National Comprehensive Cancer Network, and Japanese Society for Cancer of the Colon and Rectum. Model parameters were extracted from our retrospective data for patients with colorectal cancer who had synchronous liver and/or lung metastases and underwent curative resection. Cost‐effectiveness was assessed using an incremental cost‐effectiveness ratio for quality‐adjusted life years, with a maximum acceptable value of 5 000 000–6 000 000 JPY/33333–40 000 USD. Uncertainty in the model was assessed by probabilistic sensitivity analyses.

**Results:**

For patients with stage IV colorectal cancer after curative resection, the JSCCR‐strategy was the most cost‐effective, with an incremental cost‐effectiveness ratio of 2 888 628 JPY/19256 USD compared with the next most cost‐effective program. Probabilistic sensitivity analysis showed that the JSCCR‐strategy was most likely to be selected as the most cost‐effective (76.1%–77.9%).

**Conclusions:**

This modeling analysis found that the JSCCR‐strategy was the most cost‐effective strategy for stage IV colorectal cancer. Our findings suggest that intensive postoperative surveillance is acceptable for stage IV colorectal cancer.

## Graphical abstract

This economic evaluation used a Markov state‐transition model to analyze the cost‐effectiveness of surveillance strategies recommended by various academic societies. This study found that the surveillance program proposed by the Japanese Society for Cancer of the Colon and Rectum (JSCCR) was the most cost‐effective, with an incremental cost‐effectiveness ratio of 19 256 USD compared with the next most cost‐effective program. These findings suggest that intensive postoperative surveillance as recommended by the JSCCR is acceptable for stage IV colorectal cancer.

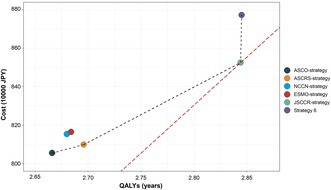

## 1 INTRODUCTION

Approximately 25% of patients with colorectal cancer (CRC) have stage IV disease with distant metastases at the time of diagnosis, and the treatment strategy selected depends on the metastatic status.^1^ The main treatment for CRC with limited distant metastasis is surgery. However, patients with stage IV CRC have a higher risk of recurrence after curative resection and poorer prognosis than those with stage I–III disease.^2^ Patients with CRC require surveillance after curative resection for early diagnosis of recurrence and therapeutic intervention. Several randomized controlled trials have examined the efficacy of intensive postoperative surveillance for CRC without distant metastases,^3^, ^4^ but the surveillance program that achieves the most favorable outcome has yet to be determined. Although various academic societies have proposed their own surveillance programs, there is currently no robust evidence to support the superiority of any strategy.

In view of the steady increase in the costs of medical care, there has been growing interest in the appropriate allocation of limited medical resources for patients with malignant diseases based on research conducted from a cost‐effectiveness perspective.^5^ For CRC, there have been many reports on the cost‐effectiveness of screening programs, multidisciplinary treatment, and chemotherapy including molecular‐targeted drugs,^6^, ^7^ but few on the cost‐effectiveness of postoperative surveillance.^8^ Patients with stage IV CRC are at higher risk of recurrence after curative resection, and the economic impact of the choice of treatment is greater for these patients than for those with stage I–III disease. However, there have been no reports on the optimal postoperative surveillance strategy for stage IV CRC from a cost‐effectiveness perspective.

The aim of this study was, therefore, to compare the cost‐effectiveness of postoperative surveillance programs recommended by the various academic societies for stage IV colorectal cancer following curative resection using a Markov modeling approach.

## 2 METHODS

This economic evaluation followed the CHEERS (Consolidated Health Economic Evaluation Reporting Standards) guideline.^9^ The study was approved by the Institutional Review Board of the National Cancer Center Hospital (code 2017‐437). Data were analyzed for the period from December 16, 2023 to March 27, 2024.

### 2.1 Model construction

**FIGURE 1:**
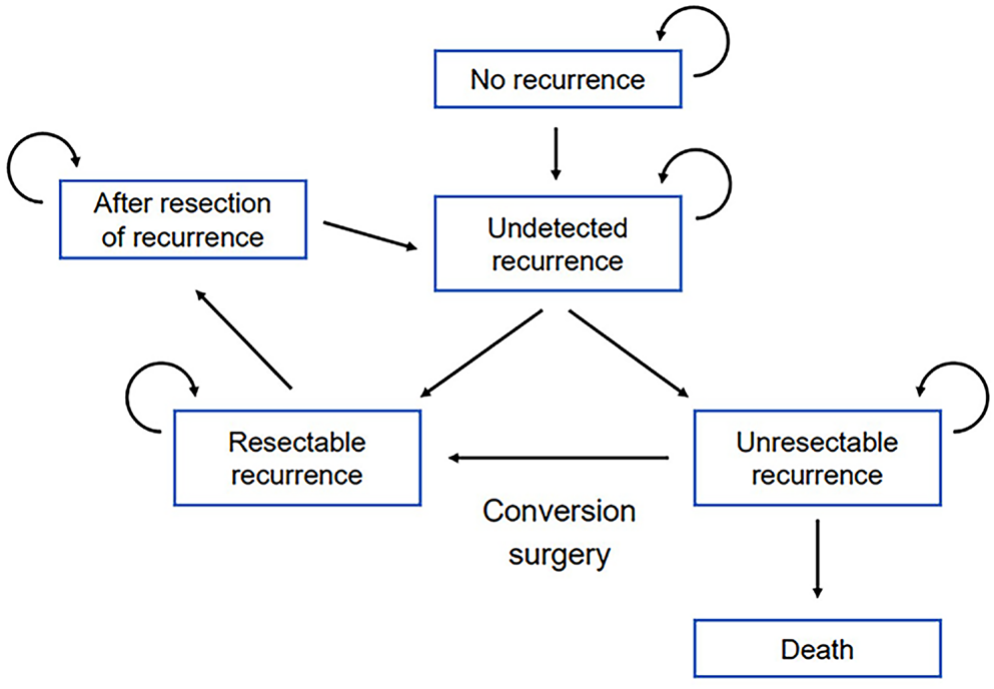
Schematic of the Markov model. A Markov state‐transition model simulating the clinical course after curative surgery for colorectal cancer is shown. The recursive arrows indicate that patients could remain in the same health status if they did not transition to another state.

We developed a Markov state‐transition model that simulated the clinical course after curative surgery for CRC (Figure 1), including diagnosis of recurrence and treatment based on resectability. We defined six discrete health states: no recurrence, undetected recurrence, resectable recurrence, unresectable recurrence, after resection of recurrence, and death. The “no recurrence” state was assigned to patients in whom curative resection of CRC was achieved. At the time when recurrence had occurred but was not yet detected, the health state shifted to “undetected recurrence.” According to the resectability of recurrent lesions at the time of examination when postoperative surveillance detected recurrence, the state changed to “resectable recurrence” or “unresectable recurrence.” “Resectable recurrence” was defined as surgery for recurrent disease performed to shift the state to “after resection of recurrence.” After surgical resection of recurrent disease, postoperative surveillance was restarted from the beginning of the surveillance strategy. The “unresectable recurrence” state was defined as a need for systemic chemotherapy. The final state was “death.” Conversion surgery for initially unresectable recurrence was treated as a shift from “unresectable recurrence” to “resectable recurrence.”

### 2.2 Model parameters

For transition probabilities and costs associated with the treatment of recurrent disease in the model, retrospective data from the National Cancer Center Hospital in Japan were used as inputs. Data of patients with stage IV CRC and liver or lung metastases who underwent R0 resection between January 2008 and December 2017 were extracted from the medical records. The distant metastatic organs were limited to the liver and lung, where the therapeutic efficacy of metastasectomy is widely accepted, because the purpose of this study was to evaluate the cost‐effectiveness of postoperative surveillance. Patients with metastatic lesions at sites other than the liver or lung, those with a history of preoperative treatment, and those with multiple cancers were excluded. Clinicopathological data, recurrence status, time to recurrence, and pattern and treatment of recurrence were evaluated. Postoperative surveillance of eligible patients was performed based on the Japanese Society for Cancer of the Colon and Rectum (JSCCR) guidelines.^10^ The transition probabilities for each health status in this model were calculated using the Kaplan–Meier method. The medical costs of postoperative surveillance and treatment of recurrence were calculated based on the Japanese medical service fee system. The costs of surgery for recurrent disease were calculated in terms of hepatectomy for liver metastases and pneumonectomy for lung metastases. The costs of chemotherapy per month were calculated for patients who were treated at our hospital from induction of chemotherapy to termination of active treatment except for palliation of symptoms. The sensitivity of each examination performed for diagnosis of recurrence and utilities (quality of life scores ranging from 0 to 1) were drawn from previous studies.^11^, ^12^

### 2.3 Postoperative surveillance program

**FIGURE 2:**
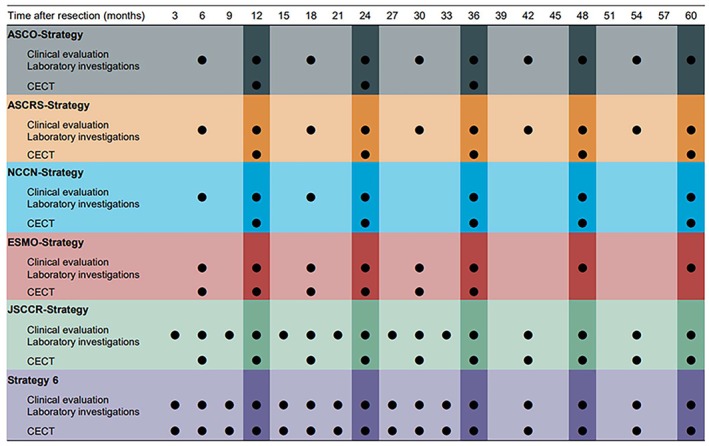
Postoperative surveillance programs evaluated in this study. The timing of the examinations in each surveillance program is shown. CECT, contrast‐enhanced computed tomography.

The surveillance programs evaluated in this study were from major guidelines published in Europe, the United States, and Japan. The frequency of examination recommended by the various surveillance programs is shown in Figure 2. From the top row of Figure 2, each strategy was a postoperative surveillance program recommended by the American Society of Clinical Oncology in 2013 (ASCO‐strategy),^13^ the American Society of Colon and Rectal Surgeons in 2021 (ASCRS‐strategy),^14^ the National Comprehensive Cancer Network in 2021 (NCCN‐strategy),^15^ the European Society of Medical Oncology in 2014 (ESMO‐strategy),^16^ and the JSCCR in 2019 (JSCCR‐strategy).^10^ In this study, strategy 6 was designed to be a more intensive surveillance program than the other five strategies. In all strategies, colonoscopy is scheduled at 1 year and 3 years postoperatively.

### 2.4 Statistical analysis

Cost‐effectiveness was analyzed from a healthcare payer's perspective by Monte Carlo simulation using TreeAge Pro (TreeAge Software Inc., Williamstown, MA). The model was analyzed for 180 cycles over 15 years, with one cycle representing 1 month. Postoperative surveillance was terminated after 5 years (60 cycles) if there was no recurrence. The analysis was performed by microsimulation with 500 000 trials. The results of the cost‐effectiveness analyses are presented as incremental cost‐effectiveness ratios (ICERs) based on quality‐adjusted life years (QALYs) as the outcome measure. ICERs were calculated as the difference in cost divided by the difference in effectiveness (QALYs) between each strategy. Strategies that were more costly and less effective than others were ruled out by simple dominance. The ICERs were evaluated for the remaining strategies. Costs and QALYs were discounted at an annual rate of 2%. In this study, costs were calculated in JPY and 2023 USD (1 USD = 150 JPY). The willingness‐to‐pay (WTP) value (i.e., the ICER threshold per QALY gained) was set at 5000000–6000000 JPY (33333–40 000 USD), which is the value commonly used in Japan.^17^

To account for the uncertainties of the parameters used in the Markov model, a probabilistic sensitivity analysis was performed for the parameters of transition probabilities, cost, examination characteristics, and utilities. In a probabilistic sensitivity analysis, the model is re‐run with each parameter being replaced with a value re‐sampled from a probability distribution. This allows for validation of the model with a population that had parameter values different from those of the original cohort, which is a valuable feature of this method. We used a beta distribution for parameters when we could acquire binary data in a population and a gamma distribution for other variables with a range of 25%. The results of the probabilistic sensitivity analysis were summarized by plotting a cost‐effectiveness acceptability curve with the WTP values for one additional QALY gained on the horizontal axis and the probability of being selected as the most cost‐effective strategy for each surveillance program on the vertical axis.

## 3 RESULTS

### 3.1 Study population and model parameters

The study included 119 patients. Their demographic and clinical characteristics are listed in Table 1. The median age was 61 years. The primary tumor was in the colon in 73 patients (61.3%) and in the rectum in 46 (38.7%). At initial diagnosis, the distant metastatic lesions were in the liver in 106 patients (89.1%), the lung in nine (7.6%), and in both the liver and lung in four (3.3%).

**TABLE 1:** Characteristics of the eligible patients.

Variable	Overall cohort
	*N* = 119
Age, mean (SD), years	60.8 (11.8)
Sex, No. (%)	
Male	69 (58.0)
Female	50 (42.0)
Primary tumor location, No. (%)	
Colon	73 (61.3)
Rectum	43 (38.7)
Pathological T status, No. (%)^a^	
pT2	4 (3.4)
pT3	85 (71.4)
pT4	30 (25.2)
Pathological N status, No. (%)^a^, ^b^	
pN0	35 (29.4)
pN1	40 (33.6)
pN2	44 (37.0)
Distribution of distant metastases, No. (%)	
Liver	106 (89.1)
Lung	9 (7.6)
Liver and lung	4 (3.3)
Adjuvant chemotherapy, No. (%)	18 (15.1)

^a^ The 8th edition of American Joint Committee on Cancer (AJCC) TNM staging system.

^b^ Lateral lymph nodes in lower rectal cancer were categorized as regional lymph nodes according to Japanese classification of colorectal cancer.

**FIGURE 3:**
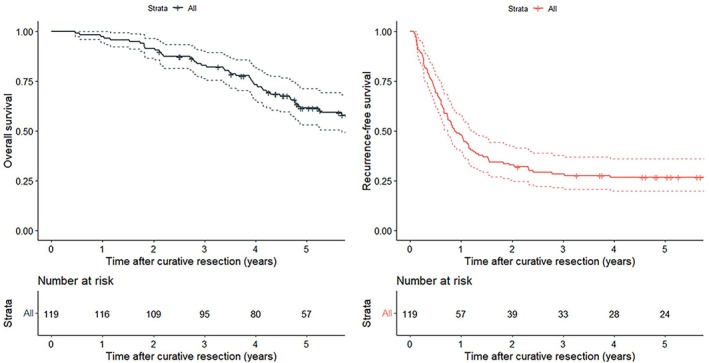
Kaplan–Meier survival curves. Kaplan–Meier survival curves for overall survival (A) and recurrence‐free survival (B) calculated from the time of curative resection in all patients.

The median follow‐up duration was 58.4 months (range, 5.6–151). The Kaplan–Meier curves for recurrence‐free survival (RFS) and overall survival (OS), which were calculated from the time of curative resection for all lesions, are shown in Figure 3. The 3‐year RFS rate was 28.5% and the 5‐year RFS rate was 26.0%; the respective 3‐year and 5‐year OS rates were 83.0% and 61.5%. During follow‐up, recurrence was detected in 88 (73.9%) of the 119 patients; the recurrent lesion was resectable in 33 cases and unresectable in 55. In the patients who underwent surgical resection for initial recurrence, the 3‐year and 5‐year RFS rates calculated from the time of surgery for recurrence were 30.3% and 23.9%, respectively. In the patients who received systemic chemotherapy for unresectable recurrence, the respective 3‐year and 5‐year OS rates calculated from the time of diagnosis of initial recurrence were 52.9% and 26.7%. The 5‐year cumulative conversion surgery rate was 19.2%. The cost of resection for hepatic metastasis was 1 950 000 JPY (13 000 USD) and that of resection for lung metastasis was 1 470 000 JPY (9847 USD). The cost of chemotherapy for unresectable recurrence was 485 000 JPY (3233 USD) per month.

The parameters used in this model are summarized in Table 2. The transition probabilities for each health status in the model were based on the survival rate in our cohort. The resection rate for recurrent disease and the conversion surgery rate are known to be strongly affected by the site and distribution of recurrence, as well as the time course after surgery.^18^ This complexity makes it challenging to express the probability as a simple time‐dependent function. To avoid overcomplication in the model, a Markov model with a fixed probability for these variables was adopted in this study. The examination costs were based on the Japanese medical service fee system. Parameters for utilities and sensitivity of examinations drawn from previous studies are shown in Table 2.

**TABLE 2:** Main input parameters in the Markov model.

Parameter	Value	Range		Distribution	Source
		Low	High		
Probability (%)					NCCH in Japan
RFS after curative resection					
1 year	47.9	39.7	57.8	Beta	
3 years	28.5	21.4	37.9	Beta	
5 years	26.7	19.8	36.0	Beta	
Resection rate at first recurrence	38.0	27.0	48.0	Beta	
Proportion of hepatectomy	70.0	59.0	82.0	Beta	
Proportion of pneumonectomy	30.0	19.0	41.0	Beta	
RFS after resection of recurrence					
1 year	66.7	52.4	84.9	Beta	
3 years	30.3	18.1	50.8	Beta	
5 years	23.9	12.9	44.2	Beta	
OS after induction of chemotherapy					
1 year	85.5	76.6	95.3	Beta	
3 years	52.9	41.0	68.4	Beta	
5 years	26.7	16.6	43.0	Beta	
Conversion rate to surgery	19.2	5.5	28.0	Beta	
Cost (JPY/USD)					Medical Service Fee System
Laboratory investigations	11 750/78.3	8437/58.7	14 062/97.9	Gamma	
CECT	38 190/255	28 643/191	47 738/319	Gamma	
Colonoscopy	15 500/103	11 625/77.3	19 375/129	Gamma	
Hepatectomy	1 950 000/13000	1 462 500/9750	2 437 500/16250	Gamma	
Pneumonectomy	1 477 000/9847	1 107 750/7385	1 846 250/12309	Gamma	
Chemotherapy, per month	485 000/3233	363 750/2425	606 250/4041	Gamma	
Utilities					Ness et al.^19^
No evidence of disease	0.74	0.56	0.93	Beta	
After resection of recurrence	0.74	0.56	0.93	Beta	
Under chemotherapy	0.25	0.19	0.31	Beta	
Sensitivity of examination					Jones et al.^20^
Clinical evaluation	0.23	0.17	0.29	Beta	
Laboratory investigations	0.58	0.43	0.70	Beta	
CECT scan	0.92	0.69	1.00	Beta	

Abbreviations: CECT, contrast‐enhanced computed tomography; NCCN, National Cancer Center Hospital; OS, overall survival; RFS, recurrence‐free survival.

### 3.2 Base case analyses

**FIGURE 4:**
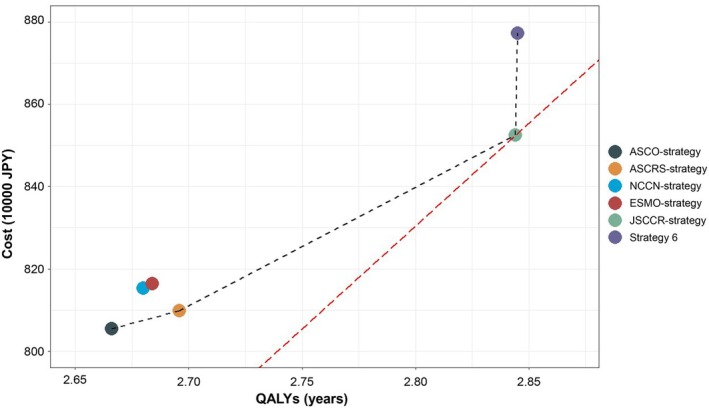
Cost‐effectiveness plane. The vertical axis shows the expected medical costs (JPY) and the horizontal axis shows the expected QALYs for each surveillance strategy in the simulation model. The black dashed line represents the efficiency frontier. The slope of the red dashed line represents the threshold of ICERs (5 000 000 JPY/QALYs). QALYs, quality‐adjusted life years; ICERs, incremental cost‐effectiveness ratios.

The results of the base case analyses are presented in Table 3 and Figure 4. ASCO‐strategy had the lowest medical cost per person (8 054 136 JPY/53694 USD) and the lowest rates of resectability of initial recurrence (34.2%), LYs (4.79 years), and QALYs (2.67 years). ASCRS‐strategy was more cost‐effective than ASCO‐strategy, with an ICER of 1 467 533 JPY/9800 USD per QALY gained. NCCN‐strategy and ESMO‐strategy were both dominated by ASCRS‐strategy, meaning that they were more costly and yielded smaller effects (LYs and QALYs). JSCCR‐strategy was the most cost‐effective surveillance program, with an ICER of 2 888 628 JPY/19256 USD per QALY gained in comparison with ASCRS‐strategy. This strategy was associated with medical costs of 8 525 679 JPY/56838 USD per person, LYs of 5.07 years, and QALYs of 2.84 years. Strategy 6, the most intensive surveillance program in this study, had higher medical costs but demonstrated similar effects to JSCCR‐strategy. Compared with JSCCR‐strategy, the ICER of strategy 6 was 246 833 000 JPY/1645000 USD per QALY gained, which exceeded the WTP threshold of 5 000 000–6 000 000 JPY/33333–40 000 USD per QALY gained.

**TABLE 3:** Base‐case cost‐effectiveness analysis.

	ASCO‐strategy	ASCRS‐strategy	NCCN‐strategy	ESMO‐strategy	JSCCR‐strategy	Strategy 6
Rate of resectability of initial recurrence	34.2%	36.1%	34.6%	35.0%	46.1%	46.5%
Medical cost per case						
JPY	8 054 136	8 098 162	8 153 181	8 163 627	8 525 679	8 772 512
USD	53 694	53 988	54 355	54 424	56 838	58 483
LYs	4.788	4.834	4.806	4.812	5.07	5.079
QALYs	2.666	2.696	2.680	2.684	2.844	2.845
ICER per QALY gained						
JPY	–	1 467 533	Dominated	Dominated	2 888 628	246 833 000
USD	–	9800	Dominated	Dominated	19 256	1 645 000

Abbreviations: ICER, incremental cost‐effectiveness ratio; LYs, life‐years; QALYs, quality‐adjusted life‐years.

### 3.3 Sensitivity analysis

**FIGURE 5:**
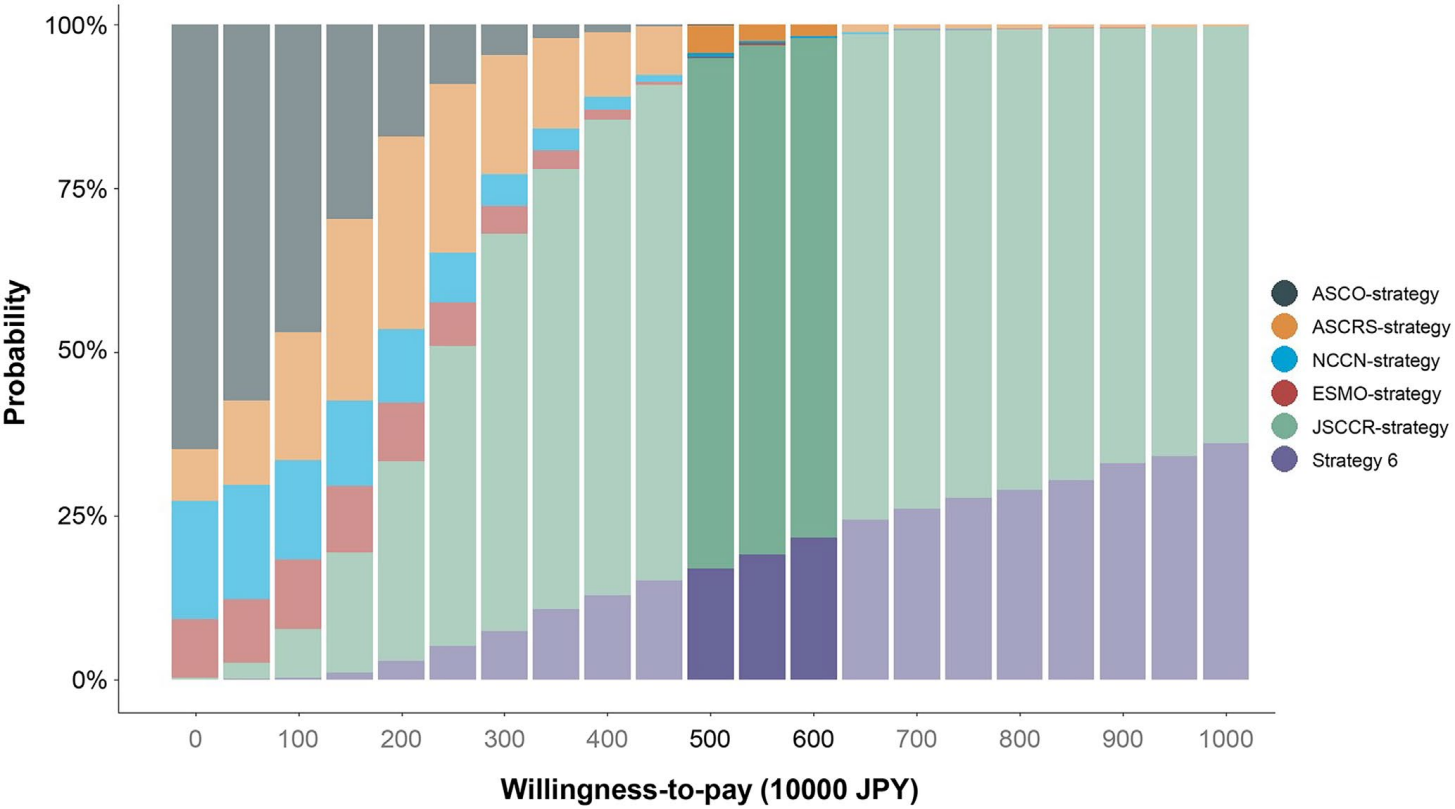
Cost‐effectiveness acceptability curve. The result of probabilistic sensitivity analysis is shown. The vertical axis shows the probability of each strategy being the most cost‐effective and the horizontal axis shows the willingness‐to‐pay threshold (JPY).

Figure 5 shows the cost‐effectiveness acceptability curve based on the results of the probabilistic sensitivity analysis. The horizontal axis represents the WTP value for one additional QALY, with a range of 0–10 000 000 JPY, and the vertical axis represents the probability of each strategy being chosen as the most cost‐effective protocol. When the WTP value was set at 5000000–6000000 JPY, JSCCR‐strategy had the highest probability of being chosen as the most cost‐effective surveillance program (76.1%–77.9%).

## 4 DISCUSSION

This study identified the program proposed by the JSCCR as the most cost‐effective surveillance strategy following curative resection of stage IV CRC with liver and/or lung metastases. Our analysis was performed using a Markov state‐transition model based on clinical data, including long‐term outcomes after curative resection of stage IV CRC and the choice of treatment for recurrent lesions and medical cost data. To our knowledge, this study is the first to determine the optimal intensity of postoperative surveillance after curative resection of stage IV CRC from a cost‐effectiveness perspective. Its findings indicate that a surveillance protocol that is more intensive than JSCCR‐strategy increases the medical cost burden but does not necessarily improve outcomes (LYs and QALYs). Our probabilistic sensitivity analysis confirmed the robustness of this simulation model.

Several prospective studies have sought to determine the optimal intensity of postoperative surveillance following curative resection for CRC without distant metastases.^3^, ^4^ Some of these studies have found that an intensive surveillance program improves the rate of local intervention for recurrent disease.^3^ However, these studies have been limited to patients with stage I–III CRC, and no prospective studies have included patients with stage IV CRC. Therefore, the optimal surveillance protocol remains to be determined, despite the various recommendations. A recently published study on the cost‐effectiveness of postoperative surveillance programs for stage I–III CRC suggested that the JSCCR protocol is overly intensive and not cost‐effective.^8^ The discrepancy in our findings regarding the intensity of postoperative surveillance between stage I–III CRC and stage IV CRC may reflect differences in recurrence rates. The recurrence rate is higher in patients with stage IV CRC than in those with stage I–III CRC. Previous studies have reported 5‐year RFS rates of 66.4%–74.3% for stage III disease,^8^, ^19^ whereas the 5‐year RFS rate for stage IV disease was 26.7% in this study. Patients with stage IV CRC are approximately twice as likely to receive treatment after diagnosis of recurrence than their counterparts with stage III CRC. The impact of choice of treatment for recurrence (i.e., surgery or chemotherapy) on overall QALYs and medical costs is more pronounced in patients at high risk of recurrence because of the large number of such patients receiving treatment.

Our results for strategies recommended by various academic societies indicate that the resectability rate for initial recurrence tended to increase as the surveillance protocol becomes more intensive. This finding suggests that the intensity of the surveillance program is one of the factors that influences the resectability rate of recurrent CRC. Previous research has also shown that intensive postoperative surveillance improves the rate of local therapeutic intervention for recurrence.^3^ However, we found that the results of strategy 6, which is more intensive than JSCCR‐strategy, were almost identical to those of JSCCR‐strategy in terms of resection rate for initial recurrence, LYs, and QALYs. This finding suggests that the benefits associated with resectability of initial recurrence and long‐term outcomes may reach a plateau with increasing surveillance intensity. Even in stage IV CRC, where the risk of recurrence is high, overly intensive surveillance programs may not improve survival outcomes and lead only to increased medical costs.

As with most cost‐effectiveness analyses, the results of this study cannot be extrapolated to other countries. Regional differences in treatment strategies for metastatic CRC and healthcare systems should be considered when interpreting the results of cost‐effectiveness analyses. The 5‐year OS rate after radical resection of stage IV CRC has ranged from 50.8% to 65.0% in previous reports from Japan,^20^, ^21^ and our finding of a 5‐year OS rate of 61.5% is within this range. In contrast, a large population‐based cohort study from the United States reported a 5‐year OS rate of 33.6% following curative resection of CRC with distant metastases localized to the liver and/or lung.^22^ This inconsistency may reflect differences in the treatment approaches for stage IV CRC and recurrent diseases, including the selection criteria for curative resection of CRC with distant metastases, in the United States and Japan. In populations at high risk of recurrence, early detection by intensive postoperative surveillance may provide greater benefits than assumed in this study. Furthermore, differences in access to medical equipment across countries should be considered. Japan has the highest number of CT units per population among Organization for Economic Co‐operation and Development countries, with approximately twice as many as the United States, which has the second‐highest number per 1 000 000 inhabitants in 2020 (115.7 units in Japan vs. 42.6 units in the United States).^23^ Therefore, it is imperative that cost‐effectiveness analyses be conducted using models that reflect the healthcare landscape in each country.

This study had several limitations. First, it had a single‐center retrospective design, included a limited number of cases, and did not consider less common treatments for metastatic lesions, such as radiofrequency ablation and stereotactic body radiotherapy.^24^ Second, only patients with stage IV CRC who had liver and/or lung metastasis were eligible for inclusion. The patient population with stage IV CRC is more heterogenous than that with stage I–III CRC in terms of metastatic organ involvement and number of metastases. For example, differences in the pattern, frequency, and timing of recurrence after curative resection for stage IV CRC have been found in patients with peritoneal dissemination or metastases to non‐regional lymph nodes and those with liver and/or lung metastases.^25^, ^26^ However, in a large multicenter study, 93.9% of patients with stage IV CRC who underwent curative resection had distant metastases localized to a single organ and 80.1% had metastases to the liver or lung.^27^ The patients who were eligible for participation in the present study were consistent with the main target population of stage IV CRC expected to achieve curative resection. Finally, it should be noted that there are several issues common to all cost‐effectiveness analyses.^28^ Medical costs fluctuate over time in response to changes in selection of chemotherapy regimens and in the medical service fee system. Variations in the cost of treatment for recurrence could change the results of simulation analysis using the Markov model. There is no absolute threshold for ICER per QALY gained worldwide, so the results of simulation analyses must be interpreted on a country‐by‐country basis.^29^ The United States uses 50 000–100 000 USD/QALY and the UK uses 20 000–30 000 GBP/QALY for ICER thresholds.^30^ Compared with the 5 000 000–6 000 000 JPY/QALY in Japan, the US threshold is higher than that used in Japan. Our sensitivity analysis showed that a higher ICER threshold increased the likelihood of acceptance of intensive surveillance programs. Even in the United States, a more intensive surveillance program following curative resection of stage IV CRC may be adopted as a cost‐effective strategy in place of current practices.

## 5 CONCLUSIONS

This study found that strategy proposed by the JSCCR to be the most cost‐effective surveillance protocol after curative resection of stage IV CRC with liver and lung metastases. The results of this analysis suggest that intensive postoperative surveillance is acceptable for stage IV CRC, although overly intensive postoperative surveillance may increase medical costs without improving the prognosis.

## AUTHOR CONTRIBUTIONS

**Fumio Tsukamoto:** Conceptualization; data curation; formal analysis; investigation; validation; visualization; writing – original draft; writing – review and editing. **Shunsuke Tsukamoto:** Conceptualization; methodology; supervision; writing – original draft; writing – review and editing. **Takeharu Kato:** Investigation; writing – review and editing. **Hiroshi Nagata:** Writing – review and editing. **Yasuyuki Takamizawa:** Writing – review and editing. **Konosuke Moritani:** Writing – review and editing. **Yusuke Kinugasa:** Conceptualization; supervision; writing – review and editing. **Minoru Esaki:** Conceptualization; funding acquisition; methodology; project administration; writing – review and editing. **Yukihide Kanemitsu:** Writing – review and editing. **Ataru Igarashi:** Conceptualization; formal analysis; methodology; project administration; software; supervision; validation; writing – review and editing.

## FUNDING INFORMATION

This study was supported by the National Cancer Center Research and Development Fund (2022‐A‐24).

## CONFLICT OF INTEREST STATEMENT

The authors declare no conflict of interest for this article. Yusuke Kinugasa is an Editorial Board Member of *Annals of Gastroenterological Surgery*.

## ETHICS STATEMENT

Approval of the research protocol: The protocol for this study was approved by the Ethics Committee of the National Cancer Center Hospital (Institutional Review Board code: 2017‐437).

Informed consent: N/A.

Registry and the Registration No. of the study: N/A.

Animal Studies: N/A.
